# VO _2_ Max Improvement of 96% in a Non-Elite Recreational Athlete over 24 Months

**DOI:** 10.1055/s-0039-1688438

**Published:** 2019-04-22

**Authors:** Daniel Laury, Armin Tehrany

**Affiliations:** 1 Department of Obstetrics and Gynecology, Western University of Health, Pomona, California; 2 Department of Obstetrics and Gynecology, University of New England, Biddeford, Maine; 3 Department of Obstetrics and Gynecology, Touro College of Osteopathic Medicine Sciences, Harlem, Manhattan, New York; 4 Department of Orthopedic Surgery, Mount Sinai School of Medicine, New York, New York; 5 Manhattan Orthopedic Care, New York, New York

**Keywords:** cardiopulmonary stress test, VO _2_ max, exercise induced asthma, aerobic exercise

## Abstract

VO _2_ max is a measure of athletic performance and is generally considered an excellent health parameter for athletic performance testing. Various methods are used to generate such a result generally using a treadmill or cycle ergometer. Improvements have been shown through training. The upper limit of such improvement has been difficult to find in the literature in part because testing often starts with individuals already at a superior level of performance. As genetics may play a significant role in an individual's VO _2_ max, training can only contribute a portion of the result. Humans have a range of maximal oxygen utilization with upper limits approaching 100 mL/kg. This case report demonstrates a 96% improvement over time secondary to selective intense training. The intent was to document objectively amelioration of the VO _2_ max using a defined protocol on standardized testing equipment. This may lead to more efficient training of individuals desiring performance improvement.

VO _2_ max is a measure of the maximal amount of oxygen that can be used at the upper limits of exercise. An increase in the VO _2_ max can be seen with training; however, generally there is a decline in maximal oxygen uptake capacity over time particularly after the third decade of life. This case documents an unusually large increase in VO _2_ max over 24 months. The training protocols used in this case report are presented that may be useful to other professionals seeking to improve their patients' performance.

## Case Report

A 43-year-old, nonsmoker, Caucasian male presented in July 2007 for a voluntary baseline cardiopulmonary stress test. He felt that he was “in good shape” and wanted to obtain an objective assessment to confirm his impression. Remaining physically active, he also followed an exercise regimen of mild weight training thrice a week and indoor rock climbing once a week. Though never a competitive athlete, he did well in high school track and field events but never pursued this to a higher level. Weight was stable at approximately 165 lbs (75 kg) at a height of 71.5 inches (182 cm) with a body mass index (BMI) of 22.7 kg/m ^2^ . These parameters were unchanged over the course of the study (± 1 kg). Diet was pescetarian, he denied any alcohol or tobacco use and supplemented only with multivitamins at the beginning of the evaluation period. He was taking no medications and was under no physician's care for any medical problems. There was a possible history of exercise induced asthma which, in fact, was demonstrated through the testing but did not limit his activity.

As this was initially a personal evaluation of his health metrics, no institutional ethics or study registration/disclosures were required. The patient was tested using a bicycle ergometer protocol available at the performance laboratory. He was monitored for blood pressure, pulse, subjective intensity using a modified Borg's scale, oxygen saturation, cardiac rhythm as well as the closed loop pulmonary function testing equipment. The initial protocol involved a ramping increment of 20 W/minute increasing until exhaustion. His effort was excellent and followed the protocol assiduously. Work capacity (VO _2_ max) was calculated at 27.6 mL/kg with a normal anaerobic threshold. Total wattage was 299. The forced expiratory volume (FEV) 1% fell by 12% suggesting mild obstructive pulmonary disease. Blood pressure was escalated to 220/100 over the course of the testing. He reached 103% of maximal calculated heart rate.

Since the result was significantly discordant to his expectation, he elected to start a training regimen to see if he could improve his results on future testing. Following the suggestions of the consulting cardiopulmonary physician, a program was designed incorporating 20 minutes of jogging thrice a week keeping his heart rate at approximately 140 bpm. He also continued the 90 minutes of rock climbing once a week. Finally, thrice a week, he started weight lifting at a higher intensity level than previously for 20 minutes per session. No type of exercise was done 2 days consecutively. Diet remained the same.

After 6 months of training, a repeat testing was done on the same equipment. The ramping protocol was set at 25 W per minute. There was a similar blood pressure escalation with maximal exertion. His VO _2_ max improved to 32.9 mL/kg. This represented a 19% improvement over baseline.

Further restructuring of the training protocol included increasing the running target heart rate to between 140 and 160 bpm. Some longer runs were added as well as a few bicycle interval workouts but these were infrequent. The patient remained compliant to instructions. There was no change in the rock climbing and weight training exercises.

The third test, now 1 year later, showed a blood pressure rise to a maximum of 206/92. VO _2_ max was measured at 42 mL/kg; a 52% improvement. The ramping protocol was the same as was the equipment.

In an effort to improve his results further, additional training modifications were instituted. Though the total volume of exercise was unchanged, intensity was further increased. On the run days, 1 day per week was designated as an interval training day done in the following fashion: 2 minutes of high intensity (up to 104% of maximal calculated heart rate) followed by 2 minutes of recovery (less than 67% of maximal calculated heart rate) in an alternating fashion for 22 minutes per session. The other 2 days per week involved keeping the heart rate just under anaerobic threshold for the full 22 to 26 minutes of training. In this patient's situation that calculated out at 162 bpm. The weight training and rock climbing protocols were left unchanged. This continued for 6 months.

On the next test, on a Cardinal Health Encore 229 unit with Cardiosoft coupled to a Ergoline pedal ergometer on a 25 work watt per minute ramping protocol 18 months after initiation of evaluation, his VO _2_ max was found to be at 50.7 mL/kg/min representing a 84% improvement over baseline. Maximal blood pressure was 212/95, maximal heart rate was 181 bpm or 106% of calculated maximum and work watts were 309.

Final testing was done 6 months later after continued training without a significant change in volume or intensity. Using the same protocol, the VO _2_ max returned a value of 54.1 mL/kg/min and work wattage at 318. Over the course of the 24 months of testing, there was a 96% improvement in VO _2_ max.

## Summary and Discussion

VO _2_ max represents the maximum ability of an individual to utilize oxygen during exertion. A high VO _2_ max, therefore, correlates with better endurance during activity. While VO _2_ max is an important component of an individual's ability to excel in sports, technique, personal psychological motivators, fatigue, nutrition, etc., may also impact on performance. Therefore, it is important to place the VO _2_ max result in context.

Maximal oxygen consumption varies among different sports and individuals as measured in professional athletes. For example, VO _2_ max is lowest in shot put on one end of the spectrum and highest in cross country skiing. It is not clear if one sport attracts a specific VO _2_ max class of athletes or if sport specific training generates a specific VO _2_ max.

Peak VO _2_ max is reached by age 19 years with a reduction over time. 1 Generally, the literature suggests a 5 to 10% reduction over each subsequent decade. Genetics may play a significant component of an individual's VO _2_ max with a contribution of 0 to 50% or more. However, identical twin studies have not all been supportive of a strong hereditary effect. 2 Furthermore, even adopted family members can also have close concordancy. 3 Summarizing Bouchard et al, 4 it would appear reasonable to accept a 25% genetic contribution.

Training has been clearly shown to improve the VO _2_ max. This was likewise demonstrated in this patient. Though it tends to decrease with age (generally 1% per year), aerobic activity tends to mitigate that drop. Interestingly, maximal heart rate does not drop with continued training. 5 Weight loss will increase the VO _2_ in an individual by definition (mL/kg/min); however, in this case report, his weight was stable. Note that this patient did not use any *β* -agonists, leukotriene inhibitors, or steroid therapy though he carried a diagnosis of exercise induced asthma. Intermittent supplementation admitted to included *α* -lipoic acid, L-acetyl carnitine, coQ10, *Rhodiola rosea* , and multivitamins. Their contribution, if any, is unclear to the documented improvement but should be considered as an unknown variable.

Humans have approached a VO _2_ max of 100 mL/kg/min. Women tend to have approximately 10 to 25% lower VO _2_ max than men depending on their training, perhaps related to their increase in type I fibers, as well as smaller diameter fibers over males. 4 Thoroughbred horses may have a VO _2_ max of 180 mL/kg/min, 6 while sled dogs have been shown to have VO _2_ max in the range of 240 mL/kg/min. 7

This interesting case demonstrated a large increase in VO _2_ max demonstrated objectively over time. The training protocols were described in detail; trainers and advisors may want to try them on their subjects to see if the results are reproducible. Various protocols have been promulgated in training literature; however, anecdotal improvements have often been the norm rather than carefully documented outcomes. It is imperative to follow a known metric since speed or lap times have shown to improve despite a stable VO _2_ max. This is likely secondary to improved muscular efficiency or mechanical aids, such as newer swimsuits and biking technology amelioration. Performance is also sensitive to other influences, such as fatigue, altitude, nutrition, and equipment. Of note, approximately 4 months into the training, the patient injured his knee (likely a meniscal tear unrelated to the training and it was not surgically addressed); however, this did not affect the training or results.

Improvements in VO _2_ max have been shown in studies using various protocols, including high intensity interval training 8 , 85% maximal heart rate for 20 minutes thrice a week 9 , hypoxia techniques, 10 and combination aerobic and weight training, 11 which generally show up to 6% improvement. One research protocol increased total amount of exercise per week to 300 minutes found an increase in VO _2_ max approaching 50%. 12 The case reported here demonstrates an unusually high increase of 96% which suggests the need to further explore parameters resulting in increased performance.
